# The application of machine learning to predict genetic relatedness using human mtDNA hypervariable region I sequences

**DOI:** 10.1371/journal.pone.0263790

**Published:** 2022-02-18

**Authors:** Priyanka Govender, Stephen Gbenga Fashoto, Leah Maharaj, Matthew A. Adeleke, Elliot Mbunge, Jeremiah Olamijuwon, Boluwaji Akinnuwesi, Moses Okpeku

**Affiliations:** 1 Discipline of Genetics, School of Life Sciences, University of KwaZulu-Natal, Westville, South Africa; 2 Faculty of Science and Engineering, Department of Computer Science, Computational Intelligence and Health Informatics Research Group, University of Eswatini, Kwaluseni, Kingdom of Eswatini; Indian Institute of Technology Patna, INDIA

## Abstract

Human identification of unknown samples following disaster and mass casualty events is essential, especially to bring closure to family and friends of the deceased. Unfortunately, victim identification is often challenging for forensic investigators as analysis becomes complicated when biological samples are degraded or of poor quality as a result of exposure to harsh environmental factors. Mitochondrial DNA becomes the ideal option for analysis, particularly for determining the origin of the samples. In such events, the estimation of genetic parameters plays an important role in modelling and predicting genetic relatedness and is useful in assigning unknown individuals to an ethnic group. Various techniques exist for the estimation of genetic relatedness, but the use of Machine learning (ML) algorithms are novel and presently the least used in forensic genetic studies. In this study, we investigated the ability of ML algorithms to predict genetic relatedness using hypervariable region I sequences; that were retrieved from the GenBank database for three race groups, namely African, Asian and Caucasian. Four ML classification algorithms; Support vector machines (SVM), Linear discriminant analysis (LDA), Quadratic discriminant analysis (QDA) and Random Forest (RF) were hybridised with one-hot encoding, Principal component analysis (PCA) and Bags of Words (BoW), and were compared for inferring genetic relatedness. The findings from this study on WEKA showed that genetic inferences based on PCA-SVM achieved an overall accuracy of 80–90% and consistently outperformed PCA-LDA, PCA-RF and PCA-QDA, while in Python BoW-PCA-RF achieved 94.4% accuracy which outperformed BoW-PCA-SVM, BoW-PCA-LDA and BoW-PCA-QDA respectively. ML results from the use of WEKA and Python software tools displayed higher accuracies as compared to the Analysis of molecular variance results. Given the results, SVM and RF algorithms are likely to also be useful in other sequence classification applications, making it a promising tool in genetics and forensic science. The study provides evidence that ML can be utilized as a supplementary tool for forensic genetics casework analysis.

## Introduction

In forensic studies, human identification is achieved through genetic profiles [1]. Over the years, genetic profile determination has largely depended on autosomal Short Tandem Repeats (STRs). However, autosomal DNA usually degrades and sometimes or not always is available in forensic settings. To alleviate such setbacks, mitochondrial DNA (mtDNA) has been applied as a marker for human identification. This is evidenced by the exponential increase in mtDNA application in forensic analysis, historical investigations and genealogical research over the past years. MtDNA possesses several major favourable characteristics, including lack of recombination, a high copy number and matrilineal inheritance, thus having greater resistance to degradation [2–4] and is the most essential alternative source of genetic information. Within the mtDNA genome, hypervariable regions I and II (HVR I and HVR II) located in the control region are highly polymorphic and contain the highest levels of variations hence making them suitable for identification purposes [1]. This also makes the region amenable for inferring genetic differentiation using analytical tools. The present study will solely focus on HVR I.

Machine learning (ML) is a subset of artificial intelligence in which ML models acquire and integrate knowledge through large-scale observations and improve and extend themselves by learning new knowledge rather than being programmed with that knowledge [5]. ML models learn from patterns in given training labels without explicit instructions and then use inference to develop useful predictions [6]. Analytical model building has been automated to perform cognitive tasks related to high-dimensional data such as classification, regression, and clustering. This is achieved by applying ML algorithms that iteratively learn from problem-specific training data, which allows computers to find hidden insights and complex patterns without explicitly being programmed. For instance, ML has been applied to identify firearms [7] as part of forensic investigation. The lack of utilizing ML for forensic applications is due to several concerns; scientists argue that this approach cannot be interrogated on how it was able to produce the evidence [8], and its use for criminal investigations will lead to more miscarriages of justice. It is not surprising then that ML in forensics is not considered as a common analysis tool. Nevertheless, one cannot disregard the fact that ML is gaining ground in prediction analysis, especially in high throughput genomic data profiling, such as high throughput sequencing and large-scale gene expression profiling and may be of value in combination with other evidence during investigation. In addition to this, Leung and team [9] conducted a review on the application of ML to determine the relationship between DNA and the quantities of key molecules in the cell, with the premise cell variables associated with disease risks. Multiple studies have applied machine learning models; logistic regression, and k-nearest neighbors, and support vector machines (SVM) to obtain meaningful genomic profiling of more disease-related variants [6,10,11]. Furthermore, the application of random forest (RF) classifier in genome analysis has been increasing rapidly in many biological studies, such as gene expression, metabolomics, proteomics, and genome-wide association [12]. These studies showed that the RF method provides good accuracy, less internal examination of error, and high variable importance from mass biological data. For instance, a study by Goldstein et al. [13] applied random forest classifier for SNP discovery related to human disease in genome-wide association dataset.

As a result, this shows the potential use of ML in forensic genetic studies in such that algorithms can be trained and used to predict the ethnicity of unknown samples. However, the major questions lie in whether ML should replace traditional sequence analysis tools such as AMOVA or serve as a supplementary tool for prediction analyses. Therefore, this study aims to apply ML to predict genetic relatedness and model genetic inferences using human mtDNA HVR I sequences, and compare Waikato Environment for Knowledge Analysis (WEKA) and Python for the implementation of ML.

## Materials and methods

The algorithm below is used to run the experiment in this study. Details of each step of the algorithm are presented under the Sections: Data Collection; Statistical Analysis; Data management and Processing; File Preparation; Data Pre-processing; Data Split; ML Classification and Performance Analysis.

**Model Algorithm**

**Step 0:** Start

**Step 1:** Obtain publicly available mtDNA HVR I data in the GenBank database for three race group (Africa, Asian and Caucasian)

**Step 2:** Capture an equal number of HVR I sequences of no mixed-raced individuals for each population group to avoid biasness

**Step 3:** Is there sequences of mixed-race individual in the dataset? If Yes Go To Step 2, Otherwise Go To Step 4

**Step 4:** Rename Sequences to retain accuracy and align with MUSCLE algorithm in MEGA software version 10.1

**Step 5:** Test the genetic structure between studied samples for variation and calculate using ARLEQUIN software version 3.5

**Step 6:** Validate genetic information using AMOVA

**Step 7:** Infer Haplogroups from each sample using MITOMASTER

**Step 8:** Pre-process the data using Principal component analysis and one hot encoding (NominalToBinary) technique to transform and enhance the quality of the data

        **Step 8.1:** Data normalization using the normalize filter in WEKA to eliminate "Noise" and avoid overfitting and underfitting

        **Step 8.2:** PCA is used for eliminating patterns that are not expected to affect the output

        **Step 8.3:** One-hot encoding is used for converting categorical data to binary number

**Step 9:** Partition the dataset into Training set (80%) and Testing set (20%)

**Step 10:** Train the ML model with the training dataset

**Step 11:** Evaluate the ML model performance using cross validation (CV) and make prediction using the testing dataset.

        **Step 11.1:** Perform 5-fold CV on the training dataset

        **Step 11.2:** Carry out performance analysis of the ML models using the 20% testing set

**Step 12:** Generate and Present the results

**Step 13:** Stop

### Data collection

In this study, the focus was obtaining HVR I sequences from individuals that belonged to either one of the following three race groups: Africans, Asians and Caucasians. Population groups including Kenya, Nigeria, China, India, Britain and Canada were selected as they are classified groups containing abundant information. There are specific mtDNA databases however, the vast majority of them are not reservoirs for sequences in comparison to National Center for Biotechnology Information (NCBI) [14]. Therefore, publicly available mtDNA HVR I data in the GenBank database was used to estimate the predictability of ethnicity (https://www.ncbi.nlm.nih.gov/genbank/). To avoid any biasness, an equal number of HVR I sequences were obtained for each population group. It was ensured that no sequences of mixed-race individuals were included in the dataset as this would have an adverse effect on the accuracy of the results and study. In total, 270 HVR I sequences were used as the dataset in this study (Table 1).

**Table 1:** Accession numbers of HVR I sequences retrieved from GenBank database.

Race Group	Population Group	Sample Size	Accession Numbers
African	Kenya	45	U93965.1—U94009.1
	Nigeria	45	U94059.1—U94104.1
Asian	China	45	AY053022.1—AY053067.1
	India	45	AJ235037.1—AJ235082.1
Caucasian	Britain	45	DQ191964.1 –DQ192009.1
	Canada	45	AF186706.1—AF186751.1

### Statistical analysis

Sequences were appropriately renamed (e.g. African 1) in order to retain accuracy and aligned with MUSCLE algorithm in MEGA software version 10.1 [15]. The genetic structure between the studied samples were tested for variation and calculated using ARLEQUIN software version 3.5 [16]. AMOVA is a common method for sequence analysis and was used in this study to validate genetic information. Haplogroups were inferred for each sample in the dataset using MITOMASTER in order to determine whether mtDNA can assign unknown samples to a geographic origin. MITOMASTER is an mtDNA sequence analysis tool available on Mitomap (https://www.mitomap.org).

### Data management and processing

**Fig 1:**
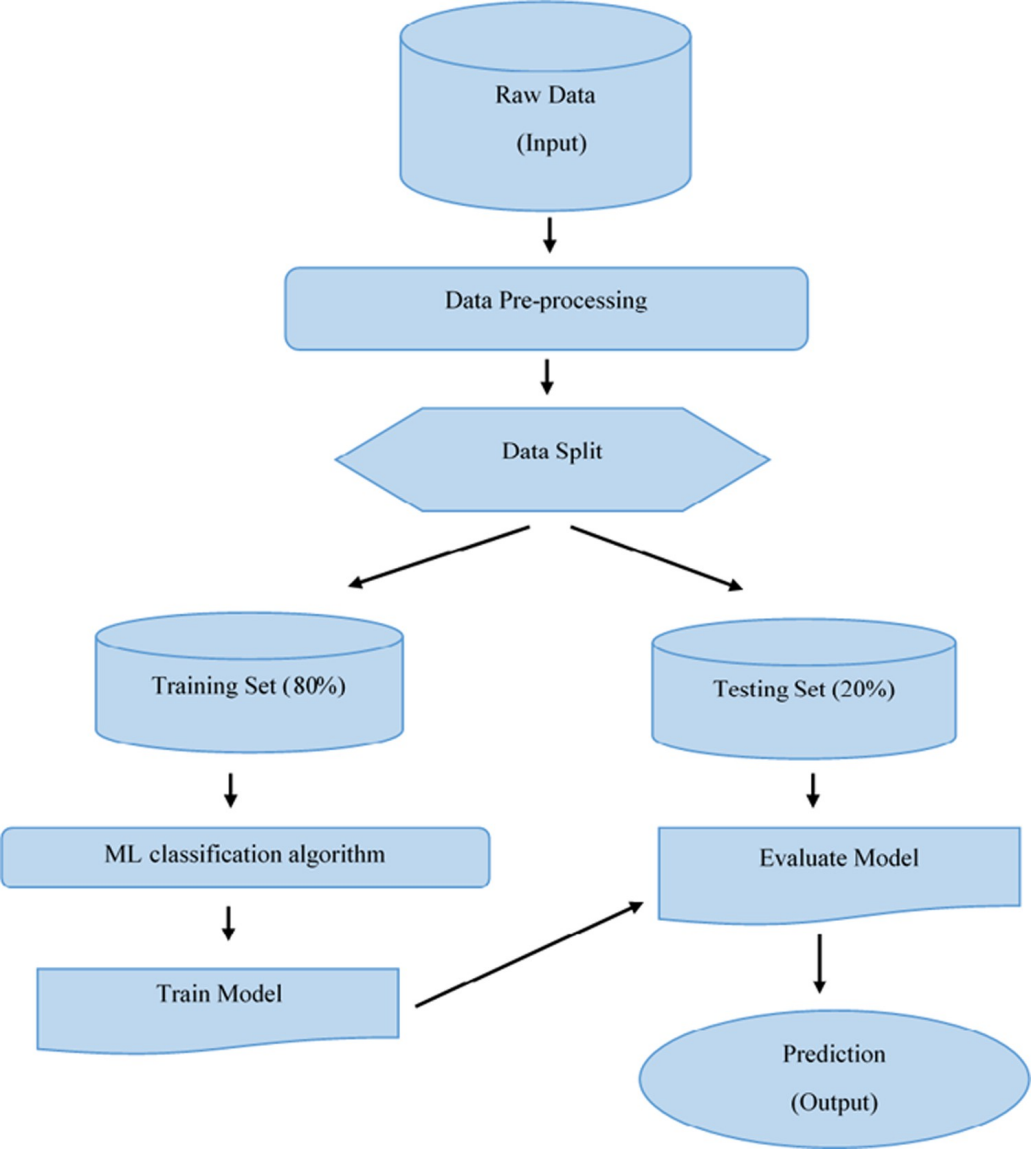
Framework for machine learning. Data is collected from database and undergo data pre-processing techniques such as one-hot encoding to transform and enhance the quality of the data. The resulting data is split into a training and testing set. The training set is used to train the ML model while the testing set is used to evaluate the model and make predictions.

The ML algorithms used in this study are Support vector machine (SVM), Random forest (RF), Linear discriminant analysis (LDA) and Quadratic discriminant analysis (QDA). Python and WEKA were chosen to evaluate the performance metrics on the dataset as they are some of the most commonly used software tools for ML. Consequently, in this paper to evaluate the ML algorithms model, we used the classification accuracy, precision, recall and f1 score performance metrics. One hot encoder and principal component analysis were used for pre-processing. The ML model was trained with Python 3.6 notebook and WEKA software version 3.8.4. The workflow for ML that was used in the present study can be seen in Fig 1.

#### File preparation

WEKA is an ML software written in JAVA that was developed by the University of Waikato, New Zealand [17,18]. The WEKA workbench is described as a collection of ML algorithms and data pre-processing tools that can be applied directly to a dataset. WEKA requires standard ARFF (Attribute Relation File Format) datasets. This is a text file that describes a list of instances sharing a set of attributes. Therefore, the datasets were converted to ARFF files for WEKA implementation. (https://www.cs.waikato.ac.nz/ml/weka/). Whereas the dataset was fed into Python as CSV files.

#### Data pre-processing

Data pre-processing is a stage in ML used to transform raw data into a more useful format. Principal component analysis and one hot encoding (NominalToBinary) was used for the pre-processing step through the use of WEKA and Python. PCA is used for eliminating patterns that are not expected to affect the output [19] while one-hot encoding is used for converting categorical data to binary number [5].

Given that *m* is the number of samples (HVR I sequences) and *n* is the number of variables in consideration, *m* can be represented as an *m* x *n* matrix. It was assumed that the sample mean for each variable is 0. By projecting *m* onto *n* new axes, it yields to **Y** = **XP** which represents the transformed dataset, where P is the orthogonal matrix whose columns represent the principal components (PCs) of the new subspace. PCs are the vectors that define the *n* new axes. This technique finds a **P** such that the sample covariance matrix of the *n* new variables defined by the PCs is a diagonal matrix containing the eigenvalues in Eq (1):

$$\sum_{Y}=\frac{1}{m}Y^{T}Y=\frac{1}{m}({XP)}^{T}XP=P^{T}\sum_{X}P=D$$ (1)

Where **D** is the diagonal matrix, ∑_**X**_ and ∑_**Y**_ represent the sample covariance matrices of the original and new variables respectively. PCA is a dimensionality reduction technique in which only ***K*** (the number of groups a given data is to be split into) and *n* (variables) are kept for further analysis [19]. For PCA, WEKA did not detect any outliers (mismatches) in the dataset, whereas Python did.

Normalization is an integral part of data preparation as it eliminates any ‘noise’, which is any irrelevant information or randomness in a given dataset. Overfitting occurs when a model familiarizes itself with the detail and noise in the training data to the extent that it negatively impacts the performance of the model on the new data. Underfitting refers to a model that can neither model the training data nor generalize to new data. Due to this, the dataset needs to be normalized to avoid overfitting and underfitting. In this study, we normalized the dataset by choosing the normalize filter in WEKA [17,18] and applying it to the dataset. The dataset was normalized to the default range of 0–1. Whereas for Python, a bag of words (BoW) was employed to convert text data into features and features into vectors. This approach gets rid of the unstructured data and noise from the text data in ML and it was applied to the study [20].

#### Data split

For both WEKA and Python, the dataset was split into 80% training set and 20% testing set. Cross-validation (CV) is a common method used to evaluate the performance of ML algorithm models [21]. In this study, ***K*** was determined by performing CV. In standard ***K***-fold CV, the data is split into subsets, called folds. A 5-fold CV was performed on the training dataset, this means that the data was split into 5 equal parts (folds), hence ***K*** = 5. The training set was trained and optimized the models, and the 20% testing set was used to evaluate the performance of the ML models.

#### Validating the performance of ML classification algorithms

To validate the ML results obtained from the initial dataset, all four algorithms’ performance was evaluated using a new independent dataset (Table 2) on both WEKA and Python. A new dataset was chosen as the classification algorithms have already been trained and evaluated with the first dataset (Table 1), hence the system is already familiar with them, and so this would not be an accurate representation of how the algorithm performs. The performance of the ML classification algorithms was also conducted on a separate new dataset (dataset 2) to avoid any biasness and for comparison purposes.

**Table 2:** Accession numbers of HVR I sequences retrieved from GenBank for dataset 2.

Race Group	Population Group	Sample Size	Accession Numbers
African	Ethiopia	45	FJ888018.1 -FJ888063.1
	Niger	45	U94112.1-U94157.1
Asian	Japan	45	AB241863.1—AB241908.1
	Korea	45	FJ494015.1—FJ494050.1
Caucasian	Germany	45	U54466.1 –U54302.1
	Russia	45	AF448676.1—AF448721.1

### Machine learning classification algorithms

Classification is a supervised learning approach in which algorithms learn from the input data and then uses this to make predictions (output). In this section, we studied four ML classification algorithms to infer genetic relatedness and HVR I sequences (dataset) was used to evaluate the overall performance of these algorithms.

#### Support vector machines

SVM is a binary classification algorithm that classifies data and separates the two classes by constructing an operating separating hyperplane (OSH) [22]. The OSH is defined by a vector *w* and a scalar *b* for *i* = 1…*n*. This typically involves solving the following optimization problem [23] of Eqs 2 and 3:

Given a training set:

$${\{(x_{i,}y_{i,}}_{}){\}}_{{}_{1\leq i\leq n}},x_{i}\epsilon R^{d},y_{i}\in \{+1,-1\}$$ (2)

SVM finds the OSH by solving Eq (3):

$$\{\begin{array}{l} \;\underset{w,b}{\min}\;\frac{1}{2}\|w{\|}^{2}\\ \mathrm{with}\;y_{i}(w,x+b)\geq 1 \end{array}$$ (3)

Where, *w* and *b* are computed using the training set during model training [23], this yields to *y* in which the new samples (test set) are classified. The classifier is trained in such that +1 denotes the correct classification of sample (correct race group) and -1 denotes the incorrect classification or misclassification of sample (other race groups). We downloaded and installed LIBSVM version 1.0.3 package to perform SVM analysis in WEKA. The SVM algorithm was implemented together with a kernel. A kernel transforms an input data space into the required form and separates classes by adding more dimension to it. There are four different types of kernel functions considered in most studies and they are radial basis function (RBF), linear, polynomial and sigmoid. According to studies carried out by Akinnuwesi et al [24], Tien Bui et al [25] and Hong et al [26] discovered that SVM models with RBF function has the highest prediction capability and performance in terms of Area Under Curve (AUC), recall, precision and classification accuracy. The RBF is not also affected by local minima, therefore, this study applied RBF for SVM analysis.

The outstanding performance of the RBF kernel over the other three kernels was influenced mainly by the values of C and gamma (*γ*) in Eq (4):

$$K(x_{i,}x_{j})=exp(-\gamma {\left\|x_{i}-x_{j}\right\|}^{2}+C),\gamma >0$$ (4)

Where *K*(x_i_,x_j_) is the kernel function and C, γ are the optimization parameters and, ‖x_i_−x_j_‖ is the Euclidean distance between x_i_ and x_j_.

#### Linear and quadratic discriminant analysis

LDA performs dimensionality reduction in which it projects the input data to a linear subspace. This subspace consists of directions which maximize the separation between classes [27]. LDA assumes that both classes have common covariance matrices, which results in a linear decision boundary. The LDA equation is represented in Eq (5):

$$\delta_{k}^{L}(X)=\mu_{k}^{T}\sum^{-1}X-\mu_{k}^{T}\sum^{-1}\mu_{k}+log(\pi_{k})$$ (5)

Where **Σ** is a covariance matrix for all class of k classes and *μ*_*k*_ is the class-specific mean vector. Since LDA assumes that the covariance matrix for different *k* is the same, there are fewer parameters to estimate compared to QDA.

The Eq (6) represents QDA:

$$\delta_{k}^{Q}(X)=-\frac{1}{2}log|\sum_{k}|\frac{1}{2}{\left(X-\mu_{k}\right)}^{T}\sum_{k}^{-1}\;\left(X-\mu_{k})+log(\pi_{k}\right)$$ (6)

Where **Σ**_**k**_ is the covariance matrix for the k^th^ class and *μ*_*k*_ is the class specific mean vector. QDA differs from LDA in such that it assumes that the covariance matrix can be different for each class which yields to a quadratic decision boundary. QDA allows for more flexibility for the covariance matrix which fits the data better than LDA; however, there are more parameters to estimate.

#### Random forest

RF is an ensemble-based ML technique that consists of multiple classification and regression trees as classifiers [28]. Each classifier is generated using a random vector sampled independently from the input vector [29], and each tree casts a unit vote for the most popular class to classify an input vector. RF classifier produces multiple decision trees, using a randomly selected subset of training samples and variables. RF can be defined by Eq (7),

$${\{h(x,\theta_{k}}_{}),k=1,\cdots ,L\}$$ (7)

Where *θ*_*k*_ is an independent random vector parameter, *x* is the input data, and *k* suggests the number of decision tree in the RF [30]. Each decision tree uses a random vector as a parameter, it randomly selects the feature of samples and thereafter selects the subset of the sample data as the training set.

### Performance analysis

Micro and Macro accuracies were calculated on the WEKA and Python classification accuracy results in order to measure the overall performance of ML classification techniques; SVM, LDA, QDA and RF to infer genetic relatedness [31]. Macro-accuracy is more like an average because it gives equal weight to each class whereas Micro-accuracy includes the contributions of confusion matrices of all classes (race groups) to get an average.

Micro and Macro accuracies were calculated using Eqs (8A) and (8B):

$$Micro‐accuracy=\frac{\sum_{i=1}^{K}C_{i}}{\sum_{i=1}^{K}N_{i}}$$ (8A)

$$Macro‐accuracy=\frac{1}{K}\sum_{i=1}^{K}\frac{C_{i}}{N_{i}}$$ (8B)

Where *K* is the number of classes in the dataset, *N*_*i*_ is the number of samples in class i and *C*_*i*_ is the number of samples correctly classified by the ML algorithm.

## Results

### Analysis of molecular variance

AMOVA is a traditional sequence analysis tool and was used in the study to validate the existence of genetic information. As shown in Table 3, 75.98% of variation occurred within populations (variation between the ethnic groups present in the populations) whereas 21.23% occurred among populations (Indian, Chinese, Nigerian, Kenyan, British and Canadian) within groups. Only 2.79% of the variation occurred among race groups (African, Asian and Caucasian). The AMOVA results showed that the majority of variance came from within populations.

**Table 3:** AMOVA showing genetic variation.

Source	Degree of Freedom	Sum of squares	Variance components	Percentage Variation (%)
**Among Groups**	2	89.559	0.09735	2.79
**Among Populations within Groups**	3	108.056	0.74144	21.23
**Within Populations**	264	700.556	2.65362	75.98

The pairwise fixation index (*F*_*ST*_) values of population differentiation due to genetic structure and p-values are shown in Table 4. *F*_*ST*_ is a measure of population differentiation due to genetic structure. The *F*_*ST*_ values demonstrated that the Canadian population showed the highest variability compared to the other population groups.

**Table 4:** Pairwise fixation index (*F*_*ST*_) values of population differentiation due to genetic structure and p-values.

Population Group	Kenyan	Nigerian	Indian	Chinese	British	Canadian
**Kenyan**		+	+	+	+	+
**Nigerian**	0.05662		+	+	+	+
**Indian**	0.12008	0.11442		+	+	+
**Chinese**	0.12307	0.14796	0.07708		+	+
**British**	0.19501	0.18562	0.09061	0.10352		+
**Canadian**	0.43895	0.49032	0.45344	0.43929	0.54525	

With reference to Table 5, the haplotype diversity (Hd) for all 270 sequences was calculated to be 1.0000 +/-0.0047 SD. Nucleotide diversity was the highest in the African group with Kenyan population group being the highest followed by the Nigerian population. The lowest nucleotide diversity was evident in the Canadian population. Neutrality indices calculated by Tajima’s D and Fu’s Fs test were negative in all populations. The D-value was significantly negative in all except the British population. All of the AMOVA results confirm that genetic variation exists between race groups which allowed for the ML algorithms to model genetic inferences.

**Table 5:** Summary of the diversity and neutrality indices calculated for population groups.

Population Group	Haplotypes	Mean Pairwise Differences	Haplotype Diversity (Hd ± S.D)	Nucleotide Diversity (nd ± S.D)	Tajima’s D	Fu Fs
**Kenyan**	45	10.236782 +/- 4.807580	1.0000 +/-0.0047	0.028436 +/- 0.014861	-1.08187	-23.82678
**Nigerian**	45	6.878161 +/- 3.329239	1.0000 +/-0.0047	0.019106 +/- 0.010291	-1.46260	-25.12242
**Indian**	45	5.577011 +/- 2.755099	1.0000 +/-0.0047	0.015492 +/- 0.008516	-1.88732	-25.39716
**Chinese**	45	7.604598 +/- 3.649328	1.0000 +/-0.0047	0.015301 +/- 0.008171	-1.43432	-24.98951
**Canadian**	45	1.131034 +/- 0.756070	1.0000 +/-0.0047	0.003346 +/- 0.002489	-1.56045	-29.04221
**British**	45	3.834483 +/- 1.983182	1.0000 +/-0.0047	0.018524 +/- 0.010661	-0.69470	-25.92218

### Machine learning on WEKA

Table 6 is a summary of the 5-fold CV accuracy metrics for ML classification algorithms hybridised with PCA namely PCA-LDA, PCA-QDA, PCA-RF and PCA-SVM on each race group. PCA-SVM consistently outperformed the other three classification algorithms investigated in this study with respect to all accuracy measures (bold values). As a result, PCA-SVM is the dominant classifier over PCA-LDA, PCA-RF and PCA-QDA for the purpose of inferring genetic relatedness. All four classification algorithms showed a greater accuracy for the Caucasian race.

**Table 6:** Comparison of 5-fold CV accuracy measures on the dataset.

Race Group	Sample Size	Classification Algorithm (%)			
		PCA-LDA	PCA-QDA	PCA-SVM	PCA-RF
**African**	90	88.15	85.43	**88.58**	88.35
**Asian**	90	76.85	70.20	**83.33**	79.23
**Caucasian**	90	91.56	82.76	**94.35**	93.87
**Micro-accuracy**		88.74	84.64	**91.66**	90.08
**Macro-accuracy**		85.52	79.46	**88.75**	87.15

Since PCA-SVM was identified as the most accurate ML algorithm in WEKA (Table 5), its performance was evaluated on the same dataset with one-hot encoding, but without PCA and 5-fold CV. This is because, CV may overestimate the practical performance of the algorithm as it ignores potentially significant biases in the dataset. The results of this experiment are shown in Table 6. In this study, we are interested in the true positive rate (population groups that are correctly classified that they are from the actual race group). The diagonal bold values represent the predicted true race group as presented in Table 7. The predicted true race group accuracies for the African and Caucasian race groups displayed in Table 7 correspond to the results in Table 6. On the contrary, the predicted race group accuracy for the Asian race dropped by 10% when PCA and CV were not applied to the dataset.

**Table 7:** Confusion matrix table of the PCA-SVM test performed on the dataset without PCA and 5-fold CV.

True Race Group	Sample Size	Predicted Race Group (%)		
		African	Asian	Caucasian
**African**	90	**86.42**	5.16	8.42
**Asian**	90	3.60	**73.89**	22.51
**Caucasian**	90	2.65	5.87	**91.48**
**Micro-accuracy**	87.95%			
**Macro-accuracy**	83.93%			

Table 8 summarizes the 5-fold CV accuracy metrics for all four ML classification algorithms hybridised with PCA. Their performances were evaluated using dataset 2 (Table 2) to validate the results obtained with the initial dataset (Tables 1 and 6). It is evident by the bold values that PCA-SVM is the dominant classifier and this corresponds to the results present in Table 6. A slight increase in accuracy measures was observed in Table 8, this is could maybe be due to the ML algorithms being more familiar with the dataset as they were trained with one dataset already and hence increased their ability to classify correctly.

**Table 8:** 5-fold CV accuracy measures on dataset 2.

Race Group	Sample Size	Classification Algorithm (%)			
		PCA-LDA	PCA-QDA	PCA-SVM	PCA-RF
**African**	90	86.55	85.13	**88.41**	87.96
**Asian**	90	76.83	70.24	**84.29**	79.04
**Caucasian**	90	91.32	82.15	**94.37**	93.85
**Micro-accuracy**		88.08	83.92	**91.93**	89.88
**Macro-accuracy**		84.90	79.17	**89.02**	86.95

### Machine learning on Python

Tables 9 and 10 shows the average of the four ML classification algorithms (SVM, LDA. QDA and RF) with BoW based on the selected performance metrics. The results clearly indicate that the performance of the algorithms is affected by the implementation of PCA in such that SVM and RF and LDA performed equally without PCA as indicated by the bold values shown in Table 9. However, only RF outperformed the other algorithms when PCA was applied (Table 10). Given the results, it can be clearly seen that PCA does have a significant impact on the performance of ML algorithms. Overall, the algorithms displayed greater performance accuracy metrics with PCA than without PCA. It was observed that the RF algorithm was dominant in Python and this is evident by the bold values displayed in Table 10.

**Table 9:** Comparison of machine learning algorithms model with one hot encoder, BoW and without PCA.

Performance metrics	BoW-SVM	BoW-RF	BoW-LDA	BoW-QDA
Accuracy	**0.889**	**0.889**	**0.889**	0.722
Precision	**0.918**	**0.918**	0.889	0.801
Recall	**0.889**	**0.889**	**0.889**	0.722
F1-score	0.885	0.885	**0.889**	0.732

**Table 10:** Comparison of machine learning algorithms model with one hot encoder, BoW and PCA.

Performance metrics	BoW-PCA-SVM	BoW-PCA-RF	BoW-PCA-LDA	BoW-PCA-QDA
Accuracy	0.926	**0.944**	0.926	0.815
Precision	0.929	**0.953**	0.926	0.866
Recall	0.926	**0.944**	0.926	0.815
F1-score	0.926	**0.944**	0.926	0.820

**Fig 2:**
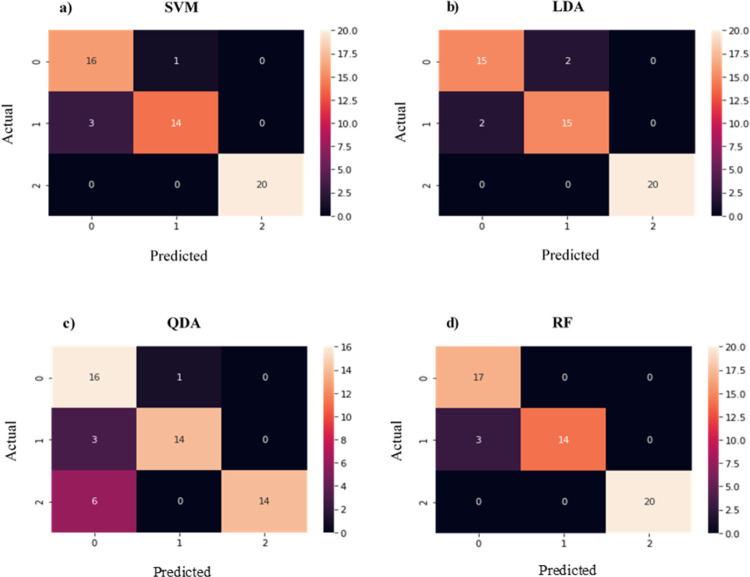
Confusion matrix results generated with one hot encoding, BoW and PCA on the dataset. Numbers 0, 1 and 2 on the X and Y axis represent the African, Asian and Caucasian race groups, respectively. The values in the matrix denote the number of correct and incorrect predictions made by classifiers: (a) Support vector machine, (b) Linear discriminant analysis, (c) Quadratic discriminant analysis and (d) Random forest.

**Fig 3:**
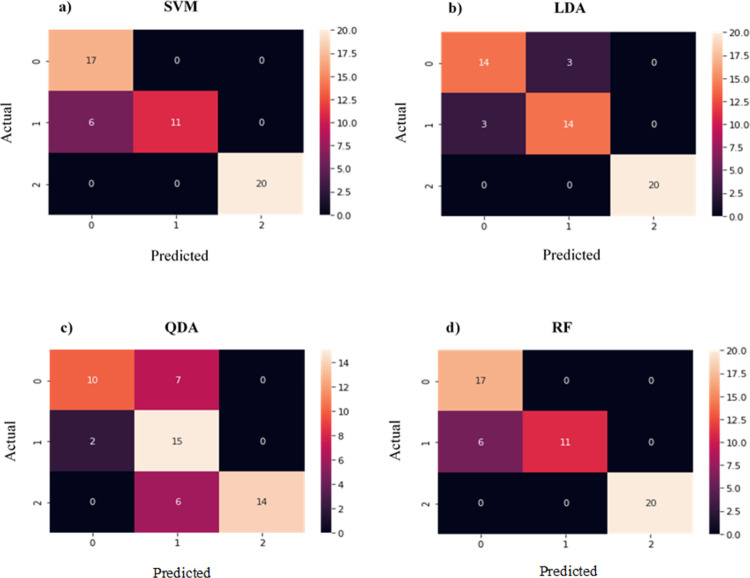
Confusion matrix results generated with one hot encoding, BoW and without PCA on the dataset. Numbers 0, 1 and 2 on the X and Y axis represent the African, Asian and Caucasian race groups, respectively. The values in the matrix denote the number of correct and incorrect predictions made by classifiers: (a) Support vector machines, (b) Linear discriminant analysis, (c) Quadratic discriminant analysis and (d) Random forest.

Tables 11 and 12 are derived from the confusion matrix on Figs 2 and 3 to indicate the correctly and incorrectly classified race groups in the mtDNA HVR I sequence dataset. As depicted in the confusion matrix results, higher accuracies were achieved when PCA was applied. Tables 10 and 11 show that QDA algorithm was the ML model most affected by PCA and the results for the African and Asian race groups confirm this (e.g. without PCA: 53.6% accuracy was achieved for the Asian race, and with PCA: 93.3% accuracy). Furthermore, the inconsistent results obtained for SVM indicate that this model was also affected by PCA (without PCA: 73.91% and 100% accuracies, and with PCA: 100% and 93.3% accuracies for the African and Asian race groups, respectively).

**Table 11:** Machine learning algorithms with one hot encoding and PCA using Python.

Race Group	Sample Size	Classification Accuracy (%)			
		BoW-PCA-SVM	BoW-PCA-RF	BoW-PCA-LDA	BoW-PCA-QDA
AFRICAN	90	84.2	85	**88.2**	64
ASIAN	90	93.3	**100**	88.2	93.3
CAUCASIAN	90	**100**	**100**	**100**	**100**
**Micro-accuracy**		95.17	97.89	94.80	88.44
**Macro-accuracy**		92.50	95.00	92.13	85.77

**Table 12:** Machine learning algorithms with one hot encoding, BoW and without PCA using Python.

Race Group	Sample Size	Classification Accuracy (%)			
		BoW-SVM	BoW-RF	BoW-LDA	BoW-QDA
AFRICAN	90	73.91	73.91	82.4	**83.3**
ASIAN	90	**100**	**100**	82.4	53.6
CAUCASIAN	90	**100**	**100**	**100**	**100**
**Micro-accuracy**		93.97	93.97	90.94	81.64
**Macro-accuracy**		91.30	91.30	88.27	78.97

Table 12 shows the classification accuracy results on Python with PCA, BoW and one hot encoder for each race group using SVM, RF, LDA and QDA algorithms from Fig 2. The Caucasian race group is classifying correctly without any error in the four classification algorithms while the classification of the African race group is with errors in the four classification algorithms model. The random forest outperforms the SVM, the LDA and QDA on the Asian race group only while LDA outperforms the SVM, random forest and the QDA on the African race group only. The random forest correctly classifies Asian and Caucasian without any error.

The classification accuracy results on Python with only one hot encoder and BoW (without PCA) for each race group using SVM, RF, LDA and QDA algorithms from Fig 3 are shown in Table 12. The Caucasian race group was able to correctly classify with any errors while the classification of the African race group obtained errors on all four classification algorithms. SVM and RF performed equally but outperformed LDA and QDA. Both SVM and RF correctly classified Asian and Caucasian race groups without any error.

Tables 13 and 14 shows the average of the four ML classification algorithms (SVM, LDA. QDA and RF) with BoW on dataset 2 based on the selected performance metrics. The results clearly indicate that the performance of the algorithms is affected by the tokenization on dataset 2 which is due to the dimension problem we discovered. The accuracy and recall as indicated by the bold values shown in Table 13 shows that BoW-SVM outperform BoW-RF, BoW-LDA and BoW-QDA while BoW-LDA outperforms BoW-RF, BoW-RF and BoW-QDA based on precision and F1-score as indicated by the bold values shown in Table 13 without PCA. However, only BoW-PCA-RF outperformed the other algorithms when PCA was applied (Table 14). Given the results, it can be clearly seen that PCA does have a significant impact on the performance of ML algorithms. Overall, the algorithms displayed greater performance accuracy metrics with PCA than without PCA. It was observed that BoW-PCA-RF algorithm was dominant in Python and this is evident by the bold values displayed in Table 14. There is a decrease in the accuracy measures as presented in Tables 13 and 14, this is due to the dimension problem we discovered in dataset 2 after tokenization. This forced us to merge the initial dataset and dataset 2 to eliminate the dimension issue which enable us to evaluate the initial dataset with the new dataset with PCA and without PCA.

**Table 13:** Comparison of machine learning algorithms model with one hot encoder, BoW and without PCA dataset 2.

Performance metrics	BoW-SVM	BoW-RF	BoW-LDA	BoW-QDA
Accuracy	**0.648**	0.47	0.548	0.337
Precision	0.433	0.351	**0.556**	0.333
Recall	**0.648**	0.47	0.548	0.337
F1-score	0.519	0.363	**0.548**	0.181

**Table 14:** Comparison of machine learning algorithms model with one hot encoder, BoW and PCA on dataset 2.

Performance metrics	BoW-PCA-SVM	BoW-PCA-RF	BoW-PCA-LDA	BoW-PCA-QDA
Accuracy	0.619	**0.652**	0.504	0.344
Precision	0.622	**0.681**	0.671	0.446
Recall	0.619	**0.652**	0.504	0.344
F1-score	0.598	**0.621**	0.397	0.19

Tables 15 and 16 summarizes the 80% training set and 20% testing set accuracy metrics for all four ML classification algorithms hybridised with PCA and without PCA. Their performances were evaluated using dataset 2 (Table 2) in order to validate the results obtained with the initial dataset (Tables 1, 9 and 10). It is evident by the bold values on Tables 13 and 14 compares with Tables 9 and 10 that there is a decrease in the performance metrics measures both without PCA and with PCA due to the dimension problem while Tables 15 and 16 compares with Tables 11 and 12 that there is a decrease in the prediction accuracy measures both without PCA and with PCA due to the dimension problem. In this scenario, for the mere fact that the ML algorithms is familiar with the initial dataset, hence their ability to classify correctly was reduced.

**Table 15:** Accuracy measures on a new independent dataset without PCA.

Race Group	Sample Size	Classification Algorithm (%)			
		BoW-LDA	BoW-QDA	BoW-SVM	BoW-RF
**African**	90	48.9	2.2	**97.8**	41.1
**Asian**	90	62.2	**98.9**	**0**	**0**
**Caucasian**	90	53.3	**0**	96.7	**100**

**Table 16:** Accuracy measures on a new independent dataset with PCA.

Race Group	Sample Size	Classification Algorithm (%)			
		BoW-PCA-LDA	BoW-PCA-QDA	BoW-PCA-SVM	BoW-PCA-RF
**African**	90	50	**100**	97.8	65.6
**Asian**	90	11.1	33.3	**35.6**	30
**Caucasian**	90	**100**	**0**	52.2	**100**

The ML algorithms on Table 15 shows the best performance on dataset 2 without PCA is the SVM with 65% accuracy, followed by the LDA with 55% and the least is the QDA with 34% for the entire population irrespective of the race. Whilst, ML algorithms on Table 16 shows the best performance on dataset 2 with PCA is the RF with over 65% accuracy, followed by the SVM with 62% and the least still remain the QDA with 34% for the entire population irrespective of the race.

### Comparison of Python and WEKA

The ML software tools employed for the evaluation of the performance metrics of the most classification accuracy in this study are Python and WEKA. As presented in Table 17, the prediction of the race group was carried out without misclassification in the BoW-PCA-RF for the Asian and Caucasian race using python but with less than 10% misclassification rate in all the classification algorithms prediction carried out in WEKA. What is responsible for the better accuracies with python than WEKA is actually the ability to create a Bag of Words (BoW) model from Sklearn in Python based on the natural language processing concept which is not possible in WEKA. Furthermore, WEKA was not able to detect any outliers during PCA, while Python did detect and remove the irrelevant attributes that would affect the results (output) of this study. Overall, Python displayed higher accuracies than WEKA, with RF algorithm obtaining 100% prediction for the Asian and Caucasian race groups with and without PCA.

**Table 17:** Race group classification accuracy (%) results from Python and WEKA.

Software tools	Classification Accuracy (%)		
	ASIAN	AFRICAN	CAUCASIAN
**PYTHON**	100(BoW-PCA-RF)	88.2(BoW-PCA-LDA)	100(BoW-PCA-RF)
**WEKA**	83.33(PCA-SVM)	88.58(PCA-SVM)	94.35(PCA-SVM)

The classification accuracies presented on Tables 15 and 16 using Python has some race group that was totally misclassified unlike on Table 8 using WEKA there was no race group with classification accuracies less than 70%. This shows that dimension problem can affect consistency of predictability on different datasets even after it has been resolved.

## Discussion

Humans can be categorized into different ethnic groups that typically reflect their geographic ancestry, using uni-parental and/or bi-parental biological markers [32]. Several studies have provided evidence on the usefulness of inferring probable race groups, ancestral and/or geographic origin from HVR I sequences [31,33], thus making mtDNA a suitable marker for ethnic affiliation prediction. The findings from these studies clearly demonstrate that although mtDNA alone does not determine one’s race but are strongly associated.

Human mitochondrial haplogroups have risen from evolutionary forces such as migration and mutation. These haplogroups have been extremely useful tools in understanding the patterns of geographical migration of human populations. Prior to modern migration, mitochondrial haplogroups were to a great extent restricted to the geographical regions of their origin and subsequent migration [31]. Similarly, race grouping humans are also reflective of geographic ancestry. Africans, Asians and Caucasians have clear geographic associations. Due to these clear associations of both mitochondrial haplogroups and race categories with geography, it is easy to expect a correlation between the two categories. S1 Table showing inferred haplogroups supports this correlation. Inferred haplogroups T, U and A consisted more of Caucasians, while B, D, M and R7 included Asians and lastly, Africans made up most of the L haplogroup (S1 Table). There is a wide correspondence between the L haplogroups and African race inferences. MtDNA represents only an exceedingly small segment of the complex mosaic of a human’s genetic ancestry and suggests that the ability to infer genetic relatedness would be limited [8]. However, genetic variation still exists between race groups and so mtDNA particularly HVR I is used to infer genetic relatedness and can assign race groups with almost 90% accuracy [31]. This high level of accuracy in predicting the genetic relatedness of unknown samples can be extremely useful in forensic investigators.

In order to understand genetic differentiation in the HVR I sequence dataset, and to lay the ground basis for comparing genetic differentiations, AMOVA was performed. The AMOVA results achieved in this study showed 75.98% variation within populations, depicting higher genetic variation within population than among populations (Table 3). Research consistently demonstrates that approximately 85% of all human genetic variation is within human populations whereas only about 15% variation exists between populations [14,34]. Hence, the results from this study support previous research findings in such that there is greater variation within races (ethnic groups) than between races. Furthermore, low to intermediate *F*_*ST*_ values (Table 4) with significant levels pointed out genetic differentiation among populations. However, the Canadian population displayed high *F*_*ST*_ values which implies a considerable degree of differentiation among populations. According to Table 5, the Kenyan and Nigerian population groups had the highest mean pairwise differences and nucleotide diversity, indicating higher degree of diversity in the African race group. These results corresponds to the reports of Campbell and Tishkoff [35] and Gomez, et al. [36] which documented high levels of genetic and phenotypic diversity present in African populations, thus making them the most diverse race in the world.

Many applications in human genetics and biology require discriminative classification of samples into groups and numerous methods for this assignment have been proposed. Over the past decade, ML has paved its way into the scientific world and has been used to good effect in several biological scenarios. In this study, four ML classification techniques (SVM, RF, LDA and QDA) were employed to determine the best (in terms of accuracy and robustness) ML classifier for genetic classification of genomic sequences. The WEKA results outlining the performance of the four classification algorithms in Table 6, highlights the dominance of SVM as a classifier. SVM consistently provided a greater accuracy level in comparison to RF, LDA and QDA for each race group (Table 6). For all four classification algorithms, the highest accuracies were observed for the Caucasian race. This meant that the Caucasian race group was more easily classified than the other two race groups. The success of SVM in WEKA suggests that it is more robust for inferring genetic relatedness as well as allocating population groups for unknown samples. These results align with the findings of studies by Lee et al. [31] and Wong et al. [37] in which SVM proved to be the dominant ML classifier. The high accuracies obtained for SVM demonstrate computationally efficiency which may be a result of using an RBF kernel that provides better accuracy with robustness.

Contrary to the WEKA results, Python identified RF as the most accurate classifier, in which it was able to achieve 100% classification accuracy for the Asian and Caucasian race groups (Table 12). It was observed that without PCA, both SVM and RF performed equally. However, when PCA was applied, SVM produced slightly higher accuracies for the African and Asian race groups (Table 10 and Fig 2). However, in Table 11 showing the performance of classification algorithms with PCA, SVM showed a significant increase and decrease in the performance accuracy for the Asian and African race, respectively. The inconsistent accuracies achieved by the SVM model were also observed with the WEKA results, in which SVM showed a 10% drop in classification accuracies for the Asian race group (Table 7) when PCA was not performed. Furthermore, QDA algorithm also showed a significant decrease in accuracy for the African race and an increase of 39.7% for the Asian race group when PCA was applied. From this, we can conclude SVM and QDA were the ML models most affected by PCA and were less efficient than LDA and RF models. The classification algorithms model accuracies with PCA outperformed those without PCA as presented in Tables 8–11.

In this study, the overall results (WEKA and Python) for all four ML models had the highest accuracy for the Caucasian race followed by the Africa race and the lowest accuracy was obtained for the Asian race (Tables 6–11). This suggests that the African race group is the more genetic diverse making them more complex to classify, and suggest a higher degree of similarity in the Caucasian race than the African and Asian race. Given the results, it is clear that Python is the better approach to analyse ML as it provided higher classification accuracies (Table 12).

Advances in genomic data reveal that early applications of ML for genetic inferences demonstrated that they outperform traditional approaches such as AMOVA [38]. Population genetics over the past five decades has been primarily focused on reconciling molecular genetic data with theoretical models like AMOVA that describe patterns of variation produced by a combination of evolutionary forces. This said, AMOVA is a powerful sequence analysis tool that has been used for many years. Comparing prediction accuracy of AMOVA as a soft tool with ML algorithm was a component explored in the present study.

Recent studies [38,39] showed that ML techniques can leverage high-dimensional data to attain far greater predictive power than traditional sequence analysis tools. Apart from the percentages generated in AMOVA indicating that there is greater variation within races, the results did not show which population group had the most and least genetic variation present, and other genetic analyses tools would be required for such output. However, in a single run, ML obtained detailed results and displayed higher accuracies. Therefore, these results support research findings which state that ML has a far greater predictive power than traditional and current sequence analysis tools [38,40]. This suggests that ML can make more precise genetic inferences than AMOVA.

Besides having a greater predictive power, time and interpretation of results were two contributing factors in this study that made ML a better sequence analysis tool than AMOVA. We found that AMOVA determination (in Arlequin software) took a longer time to generate results due to the large sequence dataset used. Although this could be different for other genetic variation tools, the present study found that ML determination took far less time to generate results using same amount of sequence data. This is in agreement with Yang, et al. [40] which mentioned that traditional sequence analysis tools can no longer handle large genomic sequence data making them inefficient in terms of computing time. Computing time for sequence analysis plays a crucial role in forensic investigations, particularly where large-scale genomic data are involved. In light of this, AMOVA computation is more redundant than ML techniques. In addition to this, there were many measures of variance produced by AMOVA and finding the most relevant result made analysis and interpretation time-consuming. Whereas for ML, the most relevant results; true group accuracies were provided, and this made analysis and interpretation simple and less laborious. Furthermore, ML results were generated in a short span of time which indicated its ability to handle large genome sequence data.

The future of genomic analyses rests in our ability to understand large and ever-growing data. ML represents a new paradigm for sequence analysis with being mainly suited for determining genetic relatedness and modelling genetic inferences in forensic studies particularly for human identification. Despite the robust and computationally efficient genetic inferences provided by ML, there are several limitations to this approach which makes it difficult to replace current sequence analysis tools. A general challenge for ML lies in its ability to make more structured genetic inferences beyond simple parameter classification [38]. Nevertheless, current developments in ML research promises future improvements to make genetic and evolutionary inferences well beyond current capabilities. Therefore, ML algorithms should be used as a supplementary sequence analysis tool for forensic applications.

## Conclusion

The results showed that PCA-SVM in WEKA and BoW-PCA-RF in Python are the most robust and accurate classifiers among compared ML algorithms with the best accuracies of 94.35% and 100%, respectively in determining genetic relatedness and modelling genetic inferences in such that it was able to classify unknown samples into race groups and infer population allocation. The success of these ML classification algorithms justify their use in genomic sequence data analysis and reiterate the need for them to be more commonly used in the field of Forensic Science particularly for human identification studies. The limitation of the present study lies in the comparison of a single genetic theory (AMOVA) with four ML algorithms. Another limitation is that only HVR I sequences were used to evaluate the performance of sequence analysis tools, other HVR regions and genomic sequence data can be used for future studies. ML has the ability to significantly aid in forensic and genetic investigations, however due to the several drawbacks mentioned above, ML cannot replace traditional sequence analysis tools but instead may serve as a supplementary tool.

## Supporting information

S1 Table Inferred haplogroups for mtDNA HVR I sequences in the dataset. (PDF)
