# Supplementary material for: The application of machine learning to predict genetic relatedness using human mtDNA hypervariable region I sequences
Source: PLoS One. 2022 Feb 18;17(2):e0263790. doi: 10.1371/journal.pone.0263790 (PMC8856515; doi:10.1371/journal.pone.0263790)
Supplement: S1 Table — (PDF) [file pone.0263790.s001.pdf]

Race Group	Population Group	Predicted Haplogroup
**African**	Kenyan1	N1a(N1a1a)
**African**	Kenyan2	M51a(M51a1)
**African**	Kenyan3	D5a(D5a3)
**African**	Kenyan4	M1a(M1a5)
**African**	Kenyan5	R0a(R0a)
**African**	Kenyan6	L2a(L2a1)
**African**	Kenyan7	M1(M1)
**African**	Kenyan8	M1a(M1a1+16093)
**African**	Kenyan9	M21b(M21b1a)
**African**	Kenyan10	R0a(R0a2b)
**African**	Kenyan11	L3i(L3i2)
**African**	Kenyan12	L2a(L2a1+143+16189 (16192)+@16309)
**African**	Kenyan13	L2a(L2a1+143+16189 (16192)+@16309)
**African**	Kenyan14	L3f(L3f1b+16292)
**African**	Kenyan15	M7c(M7c2a)
**African**	Kenyan16	M1a(M1a5)
**African**	Kenyan17	L4b(L4b2)
**African**	Kenyan18	L0f(L0f2b)
**African**	Kenyan19	L0d(L0d3b)
**African**	Kenyan20	L0f(L0f)
**African**	Kenyan21	I(I)
**African**	Kenyan22	M73b(M73b)
**African**	Kenyan23	L4b(L4b2)
**African**	Kenyan24	L2a(L2a1+16189 (16192))
**African**	Kenyan25	L5b(L5b1)
**African**	Kenyan26	L2a(L2a4b)
**African**	Kenyan27	L5b(L5b1)
**African**	Kenyan28	L3c(L3c)
**African**	Kenyan29	L3f(L3f1b4a)
**African**	Kenyan30	L0b(L0b)
**African**	Kenyan31	L0a(L0a1'4)
**African**	Kenyan32	L5b(L5b1)
**African**	Kenyan33	L5a(L5a)
**African**	Kenyan34	L3c(L3c)
**African**	Kenyan35	L3f(L3f1b4c)
**African**	Kenyan36	L0a(L0a)
**African**	Kenyan37	L0a(L0a2)
**African**	Kenyan38	L2a(L2a5)
**African**	Kenyan39	L0a(L0a2)
**African**	Kenyan40	L4b(L4b2a1)
**African**	Kenyan41	L0a(L0a1d)
**African**	Kenyan42	L3e(L3e5)
**African**	Kenyan43	L0a(L0a1'4)
**African**	Kenyan44	L0a(L0a1'4)
**African**	Kenyan45	L3f(L3f1b1a)
**African**	Nigerian1	L2b(L2b1a)
**African**	Nigerian2	L2a(L2a1c+16129)
**African**	Nigerian3	L2a(L2a1)
**African**	Nigerian4	L3e(L3e2b)

**African**	Nigerian5	L3b(L3b)
**African**	Nigerian6	L3d(L3d1c)
**African**	Nigerian7	L2a(L2a1)
**African**	Nigerian8	L3e(L3e1)
**African**	Nigerian9	L4b(L4b1a)
**African**	Nigerian10	L2a(L2a1+16189 (16192))
**African**	Nigerian11	L3e(L3e3)
**African**	Nigerian12	L1c(L1c3b2)
**African**	Nigerian13	L3e(L3e2b)
**African**	Nigerian14	L3b(L3b)
**African**	Nigerian15	L3e(L3e2)
**African**	Nigerian16	L0a(L0a1'4)
**African**	Nigerian17	L1b(L1b1a1'4)
**African**	Nigerian18	L2a(L2a1)
**African**	Nigerian19	M51a(M51a1)
**African**	Nigerian20	L1c(L1c5)
**African**	Nigerian21	L2a(L2a1a2)
**African**	Nigerian22	L3f(L3f1b+16292)
**African**	Nigerian23	L1b(L1b)
**African**	Nigerian24	L2a(L2a1)
**African**	Nigerian25	M51a(M51a1)
**African**	Nigerian26	L3e(L3e5)
**African**	Nigerian27	L3e(L3e2b)
**African**	Nigerian28	L3e(L3e5)
**African**	Nigerian29	X2(X2+225+@16223)
**African**	Nigerian30	L3d(L3d)
**African**	Nigerian31	L2a(L2a1d1)
**African**	Nigerian32	L2e(L2e)
**African**	Nigerian33	L3b(L3b)
**African**	Nigerian34	L3d(L3d)
**African**	Nigerian35	M51(M51)
**African**	Nigerian36	U5b(U5b2a1)
**African**	Nigerian37	L3b(L3b1a9a)
**African**	Nigerian38	L2a(L2a1a2)
**African**	Nigerian39	L3e(L3e3)
**African**	Nigerian40	L3e(L3e2)
**African**	Nigerian41	M51(M51)
**African**	Nigerian42	L1c(L1c3a)
**African**	Nigerian43	L3b(L3b)
**African**	Nigerian44	L2a(L2a1l3)
**African**	Nigerian45	L1b(L1b)
**Asian**	Chinese1	B5a(B5a)
**Asian**	Chinese2	B5a(B5a)
**Asian**	Chinese3	B4a(B4a4)
**Asian**	Chinese4	B4a(B4a4)
**Asian**	Chinese5	F1b(F1b1a)

**Asian**	Chinese6	M7b(M7b1a1+(16192))
**Asian**	Chinese7	D4b(D4b2a2a)
**Asian**	Chinese8	B4m(B4m)
**Asian**	Chinese9	D4j(D4j2)
**Asian**	Chinese10	M7b(M7b1a1+(16192))
**Asian**	Chinese11	B5b(B5b2)
**Asian**	Chinese12	D4o(D4o2a)
**Asian**	Chinese13	F2(F2+16291)
**Asian**	Chinese14	A7(A7)
**Asian**	Chinese15	A2(A2+(64)+@16111)
**Asian**	Chinese16	C4c(C4c1b)
**Asian**	Chinese17	N9a(N9a)
**Asian**	Chinese18	N9a(N9a11)
**Asian**	Chinese19	B4h(B4h)
**Asian**	Chinese20	M7b(M7b1a)
**Asian**	Chinese21	H1f(H1f+16093)
**Asian**	Chinese22	D4b(D4b1)
**Asian**	Chinese23	R9c(R9c1a)
**Asian**	Chinese24	B4(B4)
**Asian**	Chinese25	D5a(D5a2a)
**Asian**	Chinese26	F1b(F1b)
**Asian**	Chinese27	F1a(F1a4a1)
**Asian**	Chinese28	M7b(M7b1a1+(16192))
**Asian**	Chinese29	M10a(M10a1a1)
**Asian**	Chinese30	M7b(M7b1a1+(16192))
**Asian**	Chinese31	F1a(F1a1)
**Asian**	Chinese32	F1a(F1a1a)
**Asian**	Chinese33	M6(M6)
**Asian**	Chinese34	C4a(C4a1)
**Asian**	Chinese35	M7b(M7b1a1+(16192))
**Asian**	Chinese36	B4c(B4c1b)
**Asian**	Chinese37	F3a(F3a)
**Asian**	Chinese38	M7b(M7b1a1+(16192))
**Asian**	Chinese39	F1a(F1a)
**Asian**	Chinese40	M76a(M76a)
**Asian**	Chinese41	H1a(H1ab1)
**Asian**	Chinese42	A2u(A2u1)
**Asian**	Chinese43	B5b(B5b2a1)
**Asian**	Chinese44	B5a(B5a)
**Asian**	Chinese45	A(A)
**Asian**	Indian1	I4a(I4a1)
**Asian**	Indian2	D4e(D4e1)
**Asian**	Indian3	M2a(M2a'b)
**Asian**	Indian4	G1b(G1b)
**Asian**	Indian5	L3e(L3e2)
**Asian**	Indian6	L3h(L3h1)

**Asian**	Indian7	HV12b(HV12b1)
**Asian**	Indian8	HV4a(HV4a)
**Asian**	Indian9	M41a(M41a)
**Asian**	Indian10	W(W)
**Asian**	Indian11	M2a(M2a'b)
**Asian**	Indian12	H5(H5+16311)
**Asian**	Indian13	I4a(I4a1)
**Asian**	Indian14	M5a(M5a2a2)
**Asian**	Indian15	M6a(M6a1b)
**Asian**	Indian16	I(I)
**Asian**	Indian17	M33d(M33d)
**Asian**	Indian18	M2a(M2a1)
**Asian**	Indian19	HV4a(HV4a)
**Asian**	Indian20	U2c(U2c'd)
**Asian**	Indian21	I4a(I4a1)
**Asian**	Indian22	N(N)
**Asian**	Indian23	H15a(H15a1b)
**Asian**	Indian24	M18(M18)
**Asian**	Indian25	L3x(L3x)
**Asian**	Indian26	H1b(H1ba)
**Asian**	Indian27	U2a(U2a)
**Asian**	Indian28	S1(S1)
**Asian**	Indian29	R7a(R7a'b)
**Asian**	Indian30	U2a(U2a)
**Asian**	Indian31	R31b(R31b)
**Asian**	Indian32	L4b(L4b2b1)
**Asian**	Indian33	M54(M54)
**Asian**	Indian34	M2a(M2a1)
**Asian**	Indian35	M6a(M6a1b)
**Asian**	Indian36	U2a(U2a1)
**Asian**	Indian37	M6a(M6a1a)
**Asian**	Indian38	M31a(M31a2)
**Asian**	Indian39	M18(M18)
**Asian**	Indian40	M6a(M6a1a)
**Asian**	Indian41	M6a(M6a1a)
**Asian**	Indian42	E1a(E1a1b2)
**Asian**	Indian43	U2a(U2a)
**Asian**	Indian44	R7a(R7a'b)
**Asian**	Indian45	R7a(R7a'b)
**Caucasian**	British1	H5a(H5a4a)
**Caucasian**	British2	H5a(H5a4a)
**Caucasian**	British3	X(X)
**Caucasian**	British4	T1a(T1a)
**Caucasian**	British5	T1a(T1a)
**Caucasian**	British6	N3(N3)
**Caucasian**	British7	H3p(H3p)

**Caucasian**	British8	F4a(F4a)
**Caucasian**	British9	F2e(F2e1)
**Caucasian**	British10	H10e(H10e)
**Caucasian**	British11	X2e(X2e1)
**Caucasian**	British12	K2a(K2a11)
**Caucasian**	British13	H1(H1+16239)
**Caucasian**	British14	P5(P5)
**Caucasian**	British15	U5a(U5a1b1)
**Caucasian**	British16	R8a(R8a1a1b)
**Caucasian**	British17	T2a(T2a1b)
**Caucasian**	British18	HV0e(HV0e)
**Caucasian**	British19	I(I)
**Caucasian**	British20	W1c(W1c1)
**Caucasian**	British21	M76(M76)
**Caucasian**	British22	W(W)
**Caucasian**	British23	H1e(H1e+16129)
**Caucasian**	British24	U5a(U5a)
**Caucasian**	British25	H2a(H2a+152 16311)
**Caucasian**	British26	H2a(H2a2a1g)
**Caucasian**	British27	N1a(N1a2)
**Caucasian**	British28	T1a(T1a)
**Caucasian**	British29	T1a(T1a)
**Caucasian**	British30	U5b(U5b1b1+@16192)
**Caucasian**	British31	U5b(U5b1b1+@16192)
**Caucasian**	British32	H1a(H1a)
**Caucasian**	British33	H1a(H1a)
**Caucasian**	British34	U5a(U5a1f1)
**Caucasian**	British35	U5a(U5a1f1)
**Caucasian**	British36	U5a(U5a1+@16192)
**Caucasian**	British37	U5a(U5a1+@16192)
**Caucasian**	British38	H1a(H1a)
**Caucasian**	British39	H1a(H1a)
**Caucasian**	British40	T2b(T2b)
**Caucasian**	British41	T2b(T2b)
**Caucasian**	British42	T2b(T2b)
**Caucasian**	British43	H2a(H2a2a1g)
**Caucasian**	British44	H2a (H2a2a1g)
**Caucasian**	British45	H2a (H2a2a1)
**Caucasian**	Canadian1	A2b(A2b1)
**Caucasian**	Canadian2	A2b(A2b1)
**Caucasian**	Canadian3	A2b(A2b1)
**Caucasian**	Canadian4	A2b(A2b1)
**Caucasian**	Canadian5	A2b(A2b1)
**Caucasian**	Canadian6	D4b(D4b1a2a1)
**Caucasian**	Canadian7	A2b(A2b1)
**Caucasian**	Canadian8	A2b(A2b1)

**Caucasian**	Canadian9	A2b(A2b1)
**Caucasian**	Canadian10	A2b(A2b1)
**Caucasian**	Canadian11	A2b(A2b1)
**Caucasian**	Canadian12	A2b(A2b1)
**Caucasian**	Canadian13	A2a(A2a1)
**Caucasian**	Canadian14	D4b(D4b1a2a1)
**Caucasian**	Canadian15	A2(A2)
**Caucasian**	Canadian16	A2b(A2b1)
**Caucasian**	Canadian17	A2b(A2b1)
**Caucasian**	Canadian18	A2a(A2a1)
**Caucasian**	Canadian19	A2b(A2b1)
**Caucasian**	Canadian20	A2b(A2b1)
**Caucasian**	Canadian21	A2b(A2b1)
**Caucasian**	Canadian22	A2b(A2b1)
**Caucasian**	Canadian23	A2b(A2b1)
**Caucasian**	Canadian24	A2b(A2b1)
**Caucasian**	Canadian25	A2b(A2b1)
**Caucasian**	Canadian26	A2b(A2b1)
**Caucasian**	Canadian27	A2b(A2b1)
**Caucasian**	Canadian28	A2b(A2b1)
**Caucasian**	Canadian29	A2b(A2b1)
**Caucasian**	Canadian30	A2b(A2b1)
**Caucasian**	Canadian31	A2b(A2b1)
**Caucasian**	Canadian32	A2b(A2b1)
**Caucasian**	Canadian33	A2a(A2a1)
**Caucasian**	Canadian34	A2b(A2b1)
**Caucasian**	Canadian35	A2b(A2b1)
**Caucasian**	Canadian36	A2(A2)
**Caucasian**	Canadian37	A2b(A2b1)
**Caucasian**	Canadian38	A2b(A2b1)
**Caucasian**	Canadian39	D4b(D4b1a2a1)
**Caucasian**	Canadian40	A2b(A2b1)
**Caucasian**	Canadian41	A2b(A2b1)
**Caucasian**	Canadian42	A2(A2)
**Caucasian**	Canadian43	A2b(A2b1)
**Caucasian**	Canadian44	A2b(A2b1)
**Caucasian**	Canadian45	A2(A2)
